# Design and Analysis of A Beacon-Less Routing Protocol for Large Volume Content Dissemination in Vehicular Ad Hoc Networks

**DOI:** 10.3390/s16111834

**Published:** 2016-11-01

**Authors:** Miao Hu, Zhangdui Zhong, Minming Ni, Andrea Baiocchi

**Affiliations:** 1 State Key Laboratory of Rail Traffic Control and Safety, Beijing Jiaotong University, Beijing 100044, China; humiao@bjtu.edu.cn (M.H.); zhdzhong@bjtu.edu.cn (Z.Z.); 2 Department of Information Engineering, Electronics and Telecommunications (DIET), University of Roma Sapienza, Roma 00184, Italy; andrea.baiocchi@uniroma1.it

**Keywords:** beacon-less, end-to-end delay, link lifetime, vehicular ad hoc networks (VANETs)

## Abstract

Large volume content dissemination is pursued by the growing number of high quality applications for Vehicular Ad hoc NETworks(VANETs), e.g., the live road surveillance service and the video-based overtaking assistant service. For the highly dynamical vehicular network topology, beacon-less routing protocols have been proven to be efficient in achieving a balance between the system performance and the control overhead. However, to the authors’ best knowledge, the routing design for large volume content has not been well considered in the previous work, which will introduce new challenges, e.g., the enhanced connectivity requirement for a radio link. In this paper, a link Lifetime-aware Beacon-less Routing Protocol (LBRP) is designed for large volume content delivery in VANETs. Each vehicle makes the forwarding decision based on the message header information and its current state, including the speed and position information. A semi-Markov process analytical model is proposed to evaluate the expected delay in constructing one routing path for LBRP. Simulations show that the proposed LBRP scheme outperforms the traditional dissemination protocols in providing a low end-to-end delay. The analytical model is shown to exhibit a good match on the delay estimation with Monte Carlo simulations, as well.

## 1. Introduction

The vastly increasing interest in safety and infotainment applications has witnessed the design, experimentation and implementation of Vehicular Ad hoc NETworks (VANETs), which enables the communication among vehicles. In 1999, the United States Federal Communications Commission (FCC) allocated 75 MHz of the radio spectrum in the 5.9-GHz band for the Dedicated Short Range Communication (DSRC). In 2014, the U.S. national highway traffic safety administration announced that it had been working with the U.S. Department of Transportation on regulations that would eventually mandate vehicular communication capabilities in new light vehicles by 2017 [1].

The VANET is one of the key enabling technologies for the support of a number of applications oriented to the “intelligent transportation systems” and to the “smart cities” concept. As for the latter, urban sensing and data collection on the city environment can be supported also by using vehicles and communication protocols among them. Cameras on board vehicles are but one example of a sensor producing a high rate data stream that can be profitably propagated to vehicles moving in a region of interest. A wide scope of new real-time multimedia services, ranging from on-road safety monitoring clips to entertainment video flows, should be integrated into the design of VANETs. Predicted by Cisco, mobile video streaming has increased 70% per year recently and will take about 70% of the total network traffic by 2018 [2]. Since videos have much larger data volume compared to periodical safety beacons with tens of bytes, a more durable transmission path should be constructed to disseminate this content in VANETs. An open question is how to support large volume content delivery services between vehicles.

For VANETs, a dynamically-changing topology is one distinct difference from other networks. Many researchers preferred designing beacon-less routing protocol for VANETs because of its low overhead on the routing decision process [3,4,5]. While a beacon-less routing protocol can reduce the wireless service cost in neighbors’ information maintenance, it will introduce delay in the neighbors competition for serving as the next-hop relay. The delay problem might be more serious in transmitting large volume content, where more than one link connection needs to be established before completing the whole dissemination process. As delay appears in each round of the backbone nodes’ competition process, a more durable routing path is pursued to reduce the number of routing path construction phases. Therefore, the link lifetime should be taken into consideration on the routing protocol design for large volume content dissemination.

The traditional routing protocol always tried to extend the one-hop transmission range for reducing the end-to-end delay [3,4,5,6], leaving the path lifetime out of consideration. However, for large volume content delivery, the end-to-end path will need being re-built one or more times with high probability during the data download. Therefore, the lifetime of the constructed routing path will be crucial for the integrated content completion time. For obtaining a durable path, the designed routing protocol should increase the link lifetime and reduce the number of links for the constructed routing path, which is the core target for this paper.

In this paper, a link Lifetime-aware Beacon-less Routing Protocol (LBRP) is proposed and analyzed for large volume content dissemination in multi-hop VANETs. The LBRP protocol consists of four parts, including the SOurceVehicle (SOV) finding process, the SOV decision process, the Backbone Vehicle (BV) selection process and the BV re-selection process. Instead of periodically exchanging the beacon messages, each vehicle makes the forwarding decision based on the message header information and its current state, including the speed and position information. Besides, an analytical modeling structure is proposed to give a closed-form expression on the average delay for one constructed routing path. This is achieved by modeling LBRP routing initialization as a semi-Markov process, where the relay selection process is represented as the states transition and the transition interval denotes the beacon-less relay selection delay.

The main contributions of this paper are summarized as follows:

- To the author’s best knowledge, LBRP is the first beacon-less routing protocol designed for large volume content delivery in VANETs. To better describe the dynamic topology in the vehicular network, the path lifetime is taken into consideration in the protocol design. Apart from choosing the neighboring node nearest to the destination node, LBRP also takes the link stability factor into consideration. With this design, the constructed routing protocol can achieve a more stable routing path, with which the network throughput and the end-to-end delay performance can be enhanced.
- Based on the proposed LBRP protocol, a Markov process-based theoretical analysis structure is proposed. Compared with the centralized routing policy, the routing initialization for the beacon-less routing should be highlighted since it is one of the main sources of the delay, which is important to evaluate the performance of the proposed LBRP protocol. The proposed analytical model can estimate the transmission delay for one routing path construction.

The remainder of this paper is organized as follows. Section 2 summarizes the existing literature. Section 3 presents the system model and hypotheses. A lifetime-aware beacon-less multimedia routing protocol is proposed in Section 4. The analysis is outlined in Section 5, including the one-hop delay/distance model, the discrete Markov chain-based relay transition model and the path delay model. In Section 6, simulations are conducted to verify the proposed LBRP protocol with two other protocols. Moreover, the performance of the derived analytical results is also verified. Section 7 concludes this paper and gives some possible extension as future work.

## 2. Related Work

The routing protocol design in VANETs can be roughly divided into the “topology-based routing protocols”, “energy-aware routing protocols”, “centralized routing protocols”, “decentralized routing protocols” and “beacon-less routing protocols”. We overview the related literature for each category in the ensuing sections.

### 2.1. Topology-Based Routing Protocols

The topology-based routing protocol is one major protocol type for wireless networks, especially for the mobile ad hoc network. Several routing protocols have been defined within the working group of IETF, including Ad hoc On-demand Distance Vector routing (AODV) [7], Dynamic Source Routing (DSR) [8], Temporally Ordered Routing Algorithm (TORA) [9], Destination-Sequenced Distance-Vector routing (DSDV) [10], Topology Broadcast based on Reverse-Path Forwarding (TBRPF) [11], Optimized Link State Routing (OLSR) [12], Zone Routing Protocol (ZRP) [13], Fisheye State Routing (FSR) [14], landmark routing (LAND-MAR) [15], etc. These protocols either use a kind of flooding to detect routes on-demand or proactively maintain routing information at each node. However, these routing protocols need a big amount of signaling information exchange, especially for the proactive strategy, where the routing table should be maintained and updated dynamically. This will not be efficient to be applied in sensor networks, especially for a vehicular sensor network, and some dedicated strategy should be designed as follows.

### 2.2. Energy-Aware Routing Protocols

As one of the most important performance metrics, the energy is highlighted for most real-world deployments of wireless sensor networks (WSNs). Mottola et al. [16] proposed a number of criteria commonly used in the design of WSN protocols, where the energy-related issue is of great attention. Unfortunately, attempts to optimize energy efficiency are often in conflict with the network performance demand, e.g., the real-time observation and high throughput requirements. To increase network lifetime, Mottola et al. [17] proposed an adaptive routing algorithm to minimize the number of nodes involved in routing and balance their forwarding load. Aderohunmu et al. [18] proposed a reactive data acquisition scheme, which is built on the synergies arising from a combination of the data reduction methods and energy-efficient data compression schemes. By combining compressed sensing, data prediction and adaptive sampling strategies, the proposed scheme dramatically reduces the amount of unnecessary data transmission in the deployment for environmental monitoring and surveillance networks. Mohammed Nasr et al. [19] also proposed a clustering-based routing protocol suitable for deserts in a VANET scenario. However, the above-mentioned protocols might not be easily applied to vehicular sensor networks. As one distinct difference with the general sensor network, the vehicle as a sensor will almost be unaffected by the energy. Differently, the dynamically-changing vehicular scenario is one of the most essential issues to be considered on routing protocol design.

### 2.3. Centralized Routing Protocols

In order to maintain high Quality of Service (QoS) while reducing the wireless service cost, many content delivery applications for VANETs need an infrastructure, e.g., the Road Side Unit (RSU), to bear the central control. This kind of architecture is often named drive-thru networks [20,21,22]. Predictive Directional Greedy Routing (PDGR) [23] chooses the next-hop relay based on its present neighbors and the possible expected neighbors in the very near future. In PDGR, the packet carrier obtains the information of a possible future neighbor based on the two-hop neighbor information. Xing et al. [24,25,26] designed an adaptive video downloading scheme for throughput enhancement over the drive-thru Internet. Asefi et al. [27] proposed a multi-objective optimization framework that can enhance the video playback quality with an adaptive retransmission limit adaptation scheme. Link State-aware Geographic Routing (LSGR) protocol [28] contrived a routing metric called Expected One-transmission Advance (EOA) to improve the greedy forwarding algorithm by explicitly incorporating the link state and packet’s advance. With the consideration of the link stability from the EOA metric, the transmission efficiency can be improved by diminishing transmission failures. Slavik et al. [29] utilized the distance method to select forwarding nodes, and proposed the distribution-adaptive distance with channel quality protocol. In these schemes, it is the controlling node that handles the whole data transmission scheduling, which will leave a high load burden on the centralized side. Since the RSU deployment is still underway, the problem becomes more serious. This motivates us to focus on the decentralized scheme design.

### 2.4. Decentralized Routing Protocols

Decentralized algorithms can use the exchange of beacons to make efficient decisions for content delivery. The Greedy Perimeter Stateless Routing (GPSR) protocol [6] was proposed for wireless datagram networks that uses the positions of routers and a packer’s destination to make packet forwarding decisions. By keeping the state only about the local topology, GPSR scales better in per-router state than shortest-path and ad hoc routing protocols as the number of network destinations increases. Vinel et al. [30] demonstrated that the performance of a video-based overtaking assistant can be significantly improved by exploiting information from the beacons about any forthcoming increase in the load of the multiple access channel used. Xie et al. [31] proposed two data forwarding schemes particularly for choosing the best packet forwarder in a bi-directional multiple lane highway. Torres et al. [32] proposed an improved flooding scheme to cope with variable vehicle density situations by exchanging beacons with each other. Yu et al. [33] introduced a proactive content discovery scheme to tackle the mobility, large population and rich content challenges of VANETs.

Tripp-Barba et al. [34] proposed a new routing protocol for vehicular sensor networks that uses four different metrics, which are the distance to the destination, the traffic density, the vehicular trajectory and the available bandwidth, to make forwarding decisions for minimizing packet losses and packet delay. Chen et al. [35] proposed a multi-player game theory algorithm for intra-cluster data dissemination in vehicular sensor networks by analyzing the competitive and cooperative relationships among vehicles. Liu et al. [36] proposed a utility-based sensing task dissemination algorithm, where the sensing tasks are decomposed and offloaded to neighboring vehicles according to the utilities of the neighboring vehicles to the decomposed sensing tasks. Tian et al. [37] proposed an infrastructure-less traffic adaptive data dissemination protocol that takes into account road traffic and network traffic status for both highway and urban scenarios. Specifically, the proposed double broadcast suppression techniques in [37] can adapt efficiently to the irregular road topology.

Based on the above-mentioned work, some cross-layer strategies were proposed to synchronize the resources in different communication layers. Soldo et al. [38,39] provided a fully-distributed cross-layer solution, which leverages the properties of video coding to design a collision-resolution mechanism and the characteristics of variable bit rate traffic to efficiently exploit radio resources. Chang et al. [40,41] proposed an earliest deadline first scheme, which dynamically adjusts the priority of real-time streaming to avoid collision and introduces an admission control policy according to time constraints to provide guaranteed QoS in multi-channel environments. Xu et al. [42] presented an analytical approach that takes cross-layer factors into account and propose a new routing metric based on optimizing a queueing model that considers local loads, interferences and packet droppings. Rak et al. [43] addressed the problem of the stability of any-path communications in vehicular networks in the presence of inter-vehicle link failures. However, the dynamically-changing network topology will generate high packet transmission overhead, which motivates the adoption of beacon-less schemes.

### 2.5. Beacon-Less Routing Protocol

The beacon-less opportunistic routing scheme increases the robustness of systems for supporting transmission decisions in a completely distributed manner. The Beacon-Less Routing algorithm (BLR) presented in [44] may be the first beacon-less routing protocol that makes use of location information to reduce routing overhead. Following BLR, geographic receiver-based beacon-less approaches have been regarded as a suitable solution for forwarding video flows in VANETs. The Maximum Progress Protocol (MPP) [45] is a beacon-less protocol that comprises alternating path-discovery phases and message-delivery phases, where the path is determined by using the Dijkstra algorithm. However, MPP cannot be adapted to a vehicular network with a dynamically-changing network topology. Di Felice et al. [3] proposed a dynamic backbone-assisted protocol to support geocast communication on highway scenarios for different classes of vehicular applications. A distributed beacon-less dissemination protocol is introduced by maintaining backbone-based routes for video dissemination in multi-path V2V environments [4]. Salvo et al. [5] defined a timer-based vehicular backbone network protocol, where each vehicle can make forwarding decisions only based on the message header information, its current state and local measurements. These beacon-less routing protocols often choose the next hop according to the greedy forwarding based on the position information, regardless of the link’s quality and transmission reliability.

Apart from location information, other parameters have been also taken into account in the VANET routing protocol design [3,4,5]. Rosário et al. [46] proposed a link quality and geographical beacon-less opportunistic routing protocol for efficient video dissemination service. Yoo et al. [47] devised an efficient opportunistic relay protocol that exploits multiuser diversity and effectively copes with the dynamic channel, where the choice of a relay vehicle involves the tradeoff between the transmission range and throughput. Quadros et al. [48,49] introduced the Quality of Experience (QoE)-driven and link-quality receiver-based protocol to allow live video dissemination, where the QoE-driven parameters are offered for the relay node selection and backbone maintenance, enhancing the capacity of the system in delivering QoE-aware videos. As far as we know, the routing path lifetime has not been taken into consideration on the multihop multimedia content transmission scheme design, which will be unfolded in this paper.

## 3. System Model

In this paper, we consider vehicles moving along a linear highway segment with multiple lanes. The transmission power and the Received Signal Strength (RSS) threshold of On-Board Units’ (OBUs) equipment are the same for all vehicles. Each OBU contains a Global Position System (GPS) unit and IEEE 802.11p radio equipment. For a given vehicle, its neighboring vehicles should lie in the radio range *R*. Here, *R* is assumed to be the range within which messages can be received with a high probability of success, while reception over a distance bigger than *R* is deemed to be unreliable. This notion can take into account margins for shadowing [50,51]. The radio range can be estimated by all vehicles, e.g., by measuring the packet delivery ratio from neighbor nodes [29,52,53,54,55,56,57]. In the following design and analysis, the radio range *R* is assumed to be known in advance. To keep a relatively low control overhead, the beacon-less routing strategy is used in this paper.

For road traffic monitoring applications or video-based entertainment applications, suppose that one content SUbscribingVehicle (SUV) needs a chunk of data. The volume of the target content is large enough so that it is worth downloading it from neighboring vehicles through the VANET, rather than using the cellular network. Therefore, a SOurce Vehicle (SOV) that owns the required multimedia content should be selected as the provider instead. The chosen SOV will initialize a transmission path to the SUV and transmit the target data flow continuously.

To build a routing path from the SOV to the SUV, some intermediate vehicles, denoted as the Backbone Vehicles (BVs), would be selected to forward the target content. It should be noted that a successful multi-hop transmission depends on the vehicle density of the road. The case when at least one route exists from SOV to SUV is referred to as the connected case, while the disconnected case represents the opposite event. In the disconnected case, SUV can only send its content subscribing request to the RSUs or the cellular base stations. While in the connected case, a multi-hop transmission protocol should be designed to choose suitable vehicles as relays, which is proposed in the following (Table 1).

**Figure 1:**
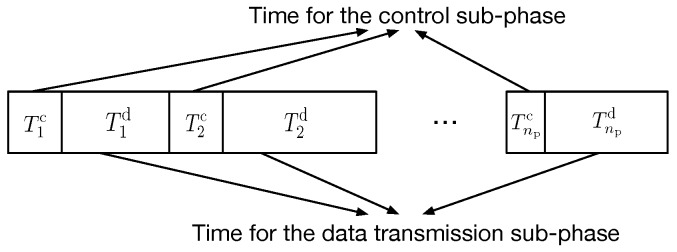
Several transmission phases for the high volume content.

Because of the dynamic network topology, the large volume content transmission can hardly finish with an unchanged routing path. Therefore, the whole content’s transmission might need more than one routing path. Each stage with one constructed routing path, defined as a transmission phase, can deliver some part of the target contents, which includes a control sub-phase and a data transmission sub-phase, as shown in Figure 1. Let $T_{i}^{c}$ and $T_{i}^{d}$ denote the consumed time for the *i*-th control sub-phase and data transmission sub-phase, respectively. Moreover, let $T_{e2e}$ denote the overall content transmission time, which is composed of transfer time for control signals and target contents. We have:

(1) $$T_{e2e}=\sum_{i=1}^{N_{p}}\left(T_{i}^{c}+T_{i}^{d}\right)=\sum_{i=1}^{N_{p}}T_{i}^{c}+T_{\mathrm{data}}\;,$$

where $T_{\mathrm{data}}$ denotes the useful data delivery time and $N_{p}$ is the number of transmission phases. It should be noted that $T_{\mathrm{data}}$ can be obtained from the target content volume and achieved link rate over the wireless channel. Let *V* be the amount of data to be delivered and *C* the average sustained physical layer link bit rate: then, $T_{\mathrm{data}}=V/C$. Then, it is $T_{i}^{c}$ and $N_{p}$ that should be determined.

Let $L_{i,j}$ denote the maximum continuous connected link holding time for the *j*-th established link of the *i*-th transmission phase, and we have:

(2) $$L_{i,j}≜\max\left\{x:s_{i,j}\left(t\right)⩾s_{\mathrm{th}},\forall t,1⩽t⩽x\right\},$$

where $s_{i,j}\left(t\right)$ denotes the RSS of the *j*-th link in the *i*-th phase at time *t* and $s_{\mathrm{th}}$ represents the minimum RSS threshold for maintaining the radio connection. Therefore, in the *i*-th transmission phase, we have the available time for delivering the target content, $T_{i}^{d}$, as:

(3) $$T_{i}^{d}=\min\left\{L_{i,1},L_{i,2},\cdots ,L_{i,H_{i}}\right\},$$

where $H_{i}$ denotes the number of hops for the *i*-th transmission phase. Hence, by taking the expectation of the equation $T_{\mathrm{data}}=\sum_{i=1}^{N_{p}}T_{i}^{d}$, we can obtain $V/C=E\left[N_{p}\right]E\left[T^{d}\right]$, where the subscript *i* of $T_{i}^{d}$ is omitted to refer to the data transmission time for a general path.

For the *i*-th transmission phase, the control sub-phase time $T_{i}^{c}$ is determined by the hop count $H_{i}$ and the control signal transmission time for each constructed link. Let $T_{L_{i,j}}$ denote the time required to set up the *j*-th link in the *i*-th transmission phase. Then, we have:

(4) $$T_{i}^{c}=\sum_{j=1}^{H_{i}}T_{L_{i,j}},$$

where the expression of $T_{L_{i,j}}$ depends on the specific dissemination scheme, and it will be derived in the following for the proposed routing protocol.

Summing it all up, the overall target content transfer time can be expressed as:

(5) $$T_{e2e}=\frac{V}{C}\left(1+\frac{E[T^{c}]}{E\left[N_{p}\right]E\left[T^{d}\right]}\right),$$

where all of the subscripts *i* in $T_{i}^{c}$ and $T_{i}^{d}$ are omitted to refer to a general case. A summary of the main symbols used throughout the paper along with their respective definitions is provided in Table 1.

## 4. Routing Protocol Design

**Figure 2:**
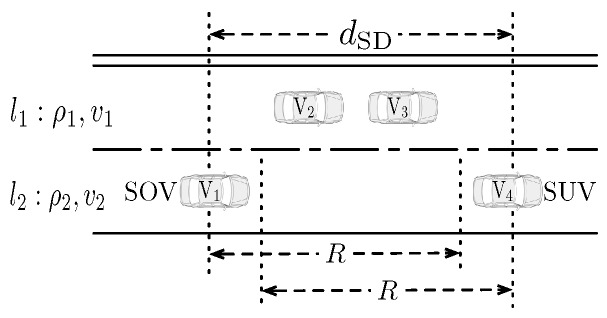
A sketch map for illustrating the designed routing protocol.

**Figure 3:**
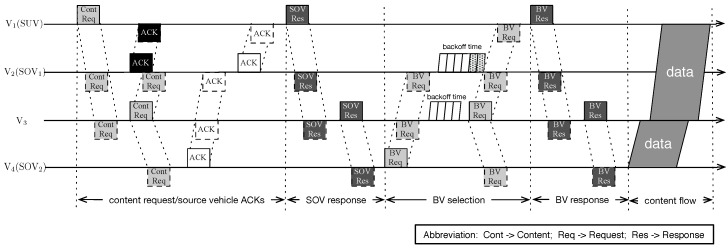
The flowchart for the designed routing scheme.

As shown in Figure 2, suppose four vehicles exist in the observed highway scenario. Based on the radio range *R*, except for the disconnected link between V${}_{1}$ and V${}_{4}$, any vehicle pair can communicate. Vehicle V${}_{1}$, denoted as the SUV, wants to obtain a specific content. Suppose that V${}_{2}$ and V${}_{4}$, defined as the SOVs, own the subscribed content. To help the SUV obtain the target content efficiently, a routing scheme is designed as illustrated in Figure 3. The designed content dissemination scheme includes the SOV finding process, the SOV decision process the BVs’ selection process and the BVs’ re-selection process. First, V${}_{1}$ broadcasts the content_request message to vehicles V${}_{2}$, V${}_{3}$ and V${}_{4}$. After receiving the requesting message, the vehicles that own the queried content, V${}_{2}$ and V${}_{4}$ in this example, transmit the ACK response back to V${}_{1}$. After comparing the characteristics of vehicles V${}_{2}$ and V${}_{4}$ as the SOV, V${}_{1}$ broadcasts the SOV_response message with the decision of the SOV selection. After receiving the SOV confirmation message, V${}_{4}$ begins the BV request process. The vehicles that receive the BV_request message will conduct the timer backoff process, and the one who wins is selected until reaching the SUV. Then, V${}_{1}$ will transmit the BV list back to the selected SOV via the BV_response message. Finally, the SOV will transmit via the selected BV chain until the constructed path breaks. These processes will be described in detail as follows.

### 4.1. SOV Finding Process

Initially, an SUV should find one suitable SOV that owns the target content and has the will to share it. The SUV will broadcast a content_request message to all vehicles within a prescribed distance defined as follows. To reduce the transmission overhead and possible delay, a Time-To-Live (TTL) value is defined to prevent long-distance flooding. For example, if TTL = 5, it means that at most, the five-hop neighbors will be queried about the target content. At each hop, the TTL value carried by the forwarded message will be decremented by one, if it is greater than zero. The position and speed information of the SUV are included in the content_request message.

A vehicle node *S* that receives the request message conducts the following verification process:

- If *S* has already received an identical content_request message before, the currently-received message will be dropped, to avoid duplicate requests.
- If instead the content_request message is new to *S*, then two alternative actions can possibly be carried out by *S*:
  – Reply: If the *S* owns the target content, it sends out an SOV_offer message, briefly denoted as an ACK, back to the SUV.
  – Forward: In case *S* does not have the requested content, if the content_request message TTL>0, the TTL is decremented by one, and the content_request message is sent forward; otherwise, if TTL=0, the received content_request message will be dropped, since the maximum transmission distance has been reached.

### 4.2. SOV Decision Process

After sending a content_request message, the SUV will wait for the ACK message responses from candidate SOVs. When the target content is of small volume, the nearest SOV is optimal to serve as the provider; consequently, the sender indicated in the first successfully received ACK response will be chosen. However, the target content is of large volume in this paper; hence, the SOV decision should be done with more than one ACK response. This is because the future network topology will affect the delivery of different parts of the large volume content, while the instantaneous topology information is enough for the delivery of small contents.

For deciding the optimal SOV, a heuristic weighting method is proposed on the observation that the transmission delay is approximately proportional to the distance between transceivers. This is because the longer the transceiver distance is, the more time will be consumed for constructing the multi-hop routing path using the timer-based routing method described in the next sub-section. Based on this observation, the weighting metric is defined as the average transceiver distance for the whole transmission phase, that is:

(6) $$w_{k}=\int_{0}^{T_{e2e}}\frac{\left(d_{k}^{0}+|v_{k}-v_{\mathrm{SUV}}|t\right)}{T_{e2e}}dt\approx d_{k}^{0}+\frac{1}{2}\left|v_{k}-v_{\mathrm{SUV}}\right|T_{\mathrm{data}},$$

where $d_{k}^{0}$ is the initial inter-vehicle distance between the SUV and the *k*-th candidate SOV and $T_{e2e}$ represents the overall content transmission time, which can be estimated as $T_{e2e}\approx T_{\mathrm{data}}$, given that we address the case of large contents in this work.

Based on the principle in Equation (6), the SOV with the minimum value of $w_{k}$ will be selected. Then, the SUV broadcasts a SOV_response message to the chosen SOV using a similar method as that in the dissemination of the content_request message.

### 4.3. BVs Selection Process

It is the chosen SOV that should be responsible for selecting BVs and transmitting the target content. First, the chosen SOV will transmit a BV_request message to all of its neighbors. Similar with that in the content_request message, a TTL value is set to limit the dissemination range. The difference is that a random backoff timer is set by each candidate BV upon receiving a new message, aiming at defining a unique winning BV that is in charge of forwarding the message [5].

Let $t_{\min}$ and $t_{\max}$ denote the minimum and maximum backoff timer values, respectively. The timer level set $T_{L_{i,j}}$ for each link will increase linearly with the values of $t_{\min}$ and $t_{\max}$. However, these two values cannot be too small, to avoid introducing a sensitive spurious forwarding problem, i.e., the occurrence of duplicated message transmissions. This problem has been discussed in many previous work, and some solutions are proposed [5,58]. Dimensioning criteria for $t_{\min}$ and $t_{\max}$ are given in [5]. In this paper, we assume that both $t_{\min}$ and $t_{\max}$ are chosen in reasonable ranges before the routing initialization process, e.g., $t_{\min}$ in the order of few milliseconds (ms), $t_{\max}$ in the order of few hundreds ms. Hence, we focus on the design of the beacon-less routing protocol for the large volume content.

With the determined values of $t_{\min}$ and $t_{\max}$, the timer $T_{L}$ is defined as (the subscript i,j in $T_{L_{i,j}}$ is omitted to refer to a generic V2V link):

(7) $$T_{L}=t_{\min}+t_{\Delta }\cdot \left(1-\epsilon \cdot \frac{L}{L_{\max}}-\left(1-\epsilon \right)\cdot \frac{D}{D_{\max}}\right),$$

where $t_{\Delta }=t_{\max}-t_{\min}$, *L* denotes the estimated link lifetime between the target vehicles, $L_{\max}$ represents the expected path lifetime for the whole route, *D* denotes the inter-vehicle distance and $D_{\max}$ denotes the maximum radio range. A candidate receiver, who can provide a more persisting link lifetime or a longer one-hop transmission distance, will have a higher chance to win the competition as a receiver. Parameter *ϵ* denotes the weighting ratio between link lifetime and the transmission distance. A higher *ϵ* will make the link lifetime more important on the routing relay selection process and vice versa. The value of *D* is no greater than $D_{\max}$ if we let $D_{\max}=R$. Differently, the value of *L* can be greater than $L_{\max}$. To guarantee that the value of *T* lies in $\left[t_{\min},t_{\max}\right]$, when $L\geq L_{\max}$, we have $T_{L}$ defined as $T_{L}=t_{\min}+t_{\Delta }\left(1-\epsilon -\left(1-\epsilon \right)D/D_{\max}\right)$.

The vehicle that receives a BV_request message will count down on the backoff time. Before the expiring of the backoff time, the vehicle will continuously listen to the channel. If it finds that the channel is occupied by an identical BV_request message with a smaller TTL value, this vehicle will terminate its timer and drop this request message (inhibition rule). Otherwise, when the timer expires, this vehicle will listen to the channel for the next slot. If the channel is idle as well, this vehicle will appoint itself as the BV and forward a new BV_request message with its own information and a minus one TTL value to continue the BV selection process, until reaching the SUV.

When the SUV receives the BV_request message, it means that the BV selection process is finished. Then, the SUV will transmit a BV_response message back to the SOV to confirm the chosen BVs information. Once the SOV receives this confirmation message, it knows that a routing path has been established between the SOV and SUV. Then, the SOV begins to transmit the target content packet by packet until a link between two consecutive BV breaks or the content transmission has finished.

### 4.4. BVs Re-Selection Process

To detect path failures, two approaches can be followed.

If the data message passing among BVs is carried out by using unicast MAC frame delivery service, MAC-level ACKs are used. Therefore, each BV gets an explicit confirmation of the reception of the data frame it forwards to the next BV or to the final destination, namely the SUV node.

If instead the data message passing exploits the broadcast service of the IEEE 802.11p interface, and MAC-level ACKs are not used. The confirmation of the correct reception of a data packet can be gained by a BV by overhearing the next BV’s transmission. As a matter of example, when BV${}_{1}$ transmits a packet to BV${}_{2}$, BV${}_{1}$ can just overhear the channel for the next transmission slot to find out whether BV${}_{2}$ has forwarded its transmitted packet. In case BV${}_{1}$ is the last BV of the chain, i.e., its next hop is the SUV node, overhearing is not effective and a network level ACK mechanism must be in place.

When one BV finds that it loses connection with the next BV, then it will generate a link_break message and transmit it back to the SOV. Once the SOV receives a link_break message, it will terminate the transmission along the current BV path and initialize a new path construction process.

## 5. Analysis of the Transmission Delay

The target of this section is to introduce an analytical model for the end-to-end transmission delay caused by the beacon-less timer setting in a route setup phase, i.e., to assess the expected value of the $T_{i}^{c}$ components of the overall content delivery time $T_{e2e}$ in Equation (1).

### 5.1. Preliminary

To describe the signal power attenuation, we adopt the path-loss model derived from highway measurements in [54]. Hence, given the sensitivity of the OBU receiver, the radio range is a constant, denoted as *R*. The vehicular traffic is assumed to be distributed along the road according to a one-dimensional Poisson point process, which has been observed in many previous works [53,59]. For the tractability of the ensuing analysis, the vehicular speed is modeled discretely, where the number of the discrete speed values is in accordance with the number of lanes. That is, in the rest of the analysis, we assume that vehicles in a specific lane keep a given speed. As shown in Figure 2, a two lane highway scenario is adopted to show the theoretical methodology. Let $\rho_{i}$ denote the traffic density (vehicles per meter) and $v_{i}$ denote the expected speed (meters per second) for the *i*-th road lane. Then, the average number of vehicles in the radio range *R* can be represented as $\lambda_{i}$, where $\lambda_{i}=\rho_{i}R$ and i∈{1,2}.

We adopt the Semi-Markov Process (SMP) for analyzing the delay issue. A continuous-time stochastic process is an SMP when the embedded state transition chain (the discrete states representing in which lane the relay vehicle is located) is a Markov chain, and the transition times are random variables, whose probability distribution function (pdf) depends on the two states between which the transition is made. The SMP can be characterized by:

(8) $$X_{0}\overset{Y_{0}}{⟶}X_{1}\overset{Y_{1}}{⟶}X_{2}\overset{Y_{2}}{⟶}\cdots ,$$

where $\left\{X_{n},n\geq 0\right\}$ is a discrete Markov chain where $X_{n}$ represents the lane state for the *n*-th BV, and $X_{n}$ can be $l_{1}$ or $l_{2}$. Transitions $\left\{Y_{n},n\geq 0\right\}$ denote the timer delay between two consecutive BVs; $Y_{n}$ depends on states $\left\{X_{n},X_{n+1}\right\}$, but it is independent of the previous timer delays $Y_{k},k<n$.

Since both $H_{i}$ and $T_{L_{i,j}}$ are random variables that can hardly yield a closed-form expression, an approximation method for the backoff timer delays is based on the expected values of the involved random variables. Based on Equation (4), we have:

(9) $$E\left[T^{c}\right]=E\left[H\right]E\left[T_{L}\right],$$

where $E[T^{c}]$ denotes the expected delay for a generic routing phase out of the $N_{p}$ ones that are carried out during the multimedia content delivery, E[H] is the average number of hops and $E[T_{L}]$ represents the expected timer delay in one V2V link. E[H] can be found as:

(10) $$E\left[H\right]=d_{\mathrm{SD}}/E\left[D_{L}\right],$$

where $d_{\mathrm{SD}}$ is the end-to-end distance between the SUV and the selected SOV, and $E[D_{L}]$ denotes the average transmission distance for each established link between two consecutive BVs.

The main goal of this section is to derive the probability distributions and mean values of $T_{L}$ in Equation (9) and $D_{L}$ in Equation (10), respectively.

The reminder of this section is organized as follows. First, given the current lane state $X_{n}$ and the next hop lane state $X_{n+1}$, the conditional distribution of one-hop link delay/distance for the intra-/inter-lane scenario, $\mathrm{Pr}\{T_{L}|X_{n},X_{n+1}\}$ and $\mathrm{Pr}\{D_{L}|X_{n},X_{n+1}\}$, are derived (Section 5.2). Based on the conditional timer distribution, the distribution of the one-hop timer delay is obtained (Section 5.3). Based on the conditioned distance distribution and the lane state transition matrix, the distribution of one-hop transmission distance is obtained (Section 5.4). Finally, based on these obtained results, we estimate the control delay time in one constructed routing path (Section 5.5).

### 5.2. One-Hop Delay/Distance for Intra-/Inter-Lane Scenario

The random variable $T_{L}$ for a generic link in a path setup phase is defined as the minimum among the backoff timer levels set by the N=n vehicle nodes reached by the path setup message issued by an elected BV node *A*. Those vehicles run their respective backoff timers to decide which one is going to forward the message to elect itself as next hop BV node *B*, as explained in Section 4.

Let $T_{j}$ denote the backoff timer level set by the *j*-th vehicle node, triggered by the reception of the path setup message issued by node *A*. Then, $T_{L}=\min\left\{T_{1},T_{2},\cdots ,T_{n}\right\}$, given that N=n vehicle nodes are running their timers at the considered hop. Note that *N* is the number of vehicle nodes within the range of *A* that have not already received the path setup message.

We will focus on the random variable $T_{L}$ conditional on the lane that *A* and *B* belong to, i.e., we define $T_{L}^{\left(1,1\right)}$ to be the random variable $T_{L}$ conditional on $X_{k}=l_{1}$ and $X_{k+1}=l_{1}$; $T_{L}^{\left(1,2\right)}$ to be the random variable $T_{L}$ conditional on $X_{k}=l_{1}$ and $X_{k+1}=l_{2}$. In the sequel, we drop the subscript *L* whenever there is no ambiguity, for the sake of simple notation. Analogously, we denote $T_{j}^{\left(1,1\right)}$ the timer of the *j*-th vehicle node (j=1,⋯,n) running its timer in the considered hop, conditional on $X_{k}=l_{1}$ and $X_{k+1}=l_{1}$.

The conditional timer delay distribution calculation will be divided into two parts: the intra-lane case for the random variable $T_{L}^{\left(1,1\right)}$ and the inter-lane case for the random variable $T_{L}^{\left(1,2\right)}$, which will be derived separately.

#### 5.2.1. One-Hop Timer Delay for the Intra-Lane Scenario

In this scenario, the link lifetime is no longer a critical problem when all of the vehicles tend to drive with identical speed. Therefore, we have the one-hop timer delay as:

(11) $$T_{j}^{\left(1,1\right)}=t_{\min}+t_{\Delta }\left(1-\epsilon \right)\left(1-\frac{D_{j}^{\left(1,1\right)}}{R}\right).$$

Since the vehicle spatial distribution follows a one-dimensional Poisson process, the vehicle position, conditional on N=n, has a uniform probability distribution in the range [0,R]; hence, the random variables $D_{j}^{\left(1,1\right)}$ are independent on one another and uniform over [0,R]. Then, we have:

(12) $$\begin{array}{cc} \mathrm{Pr}\{T^{\left(1,1\right)}\leq t|N=n\} & =1-\mathrm{Pr}\left\{T^{\left(1,1\right)}>t|N=n\right\}\\ & =1-\mathrm{Pr}\left\{T_{1}^{\left(1,1\right)}>t|N=n\right\}\cdots \mathrm{Pr}\left\{T_{n}^{\left(1,1\right)}>t|N=n\right\}, \end{array}$$

where $T_{k}^{\left(1,1\right)}∼U\left(t_{\min},t_{\min}+t_{\Delta }\left(1-\epsilon \right)\right)$. The Complementary Cumulative Density Function (CCDF) of the timer level of each receiver vehicle can be represented as:

(13) $$\mathrm{Pr}\left\{T_{k}^{\left(1,1\right)}>t|N=n\right\}=1-\left(\alpha_{1}t+\beta_{1}\right),$$

for k=1,⋯,n, and for all *t* belonging to the interval $\left[t_{\min},t_{\min}+t_{\Delta }\left(1-\epsilon \right)\right]=\left[-\beta_{1}/\alpha_{1},\left(1-\beta_{1}\right)/\alpha_{1}\right]$, where $\alpha_{1}=1/t_{\Delta }\left(1-\epsilon \right)$ and $\beta_{1}=-t_{\min}/t_{\Delta }\left(1-\epsilon \right)$. Therefore,

(14) $$\mathrm{Pr}\left\{T^{\left(1,1\right)}>t|N=n\right\}={\left[1-\left(\alpha_{1}t+\beta_{1}\right)\right]}^{n}.$$

Since the number of vehicles follows the Poisson distribution with parameter $\lambda_{1}$, the Cumulative Distribution Function (CDF) of $T^{\left(1,1\right)}$, conditional on there being at least one vehicle in the transmission range of the initiating vehicle *A*, can be represented as:

(15) $$\begin{array}{cc} \mathrm{Pr}\{T^{\left(1,1\right)}\leq t|N>0\} & =1-\mathrm{Pr}\{T^{\left(1,1\right)}>t|N>0\}\\ & =1-\sum_{n=1}^{\infty }\mathrm{Pr}\left\{T^{\left(1,1\right)}>t|N=n\right\}\mathrm{Pr}\left\{N=n|N>0\right\}\\ & =\frac{1-\exp\left(-\lambda_{1}\left(\alpha_{1}t+\beta_{1}\right)\right)}{1-\exp\left(-\lambda_{1}\right)}. \end{array}$$

holding for $t\in I_{1}≜\left[t_{\min},t_{\min}+t_{\Delta }\left(1-\epsilon \right)\right]$.

Based on the results of conditional CDF, the conditional pdfcan be represented as:

(16) $$f_{T^{\left(1,1\right)}|N>0}\left(t\right)=\frac{\lambda_{1}\alpha_{1}\exp\left(-\lambda_{1}\left(\alpha_{1}t+\beta_{1}\right)\right)}{1-\exp\left(-\lambda_{1}\right)}.$$

for $t\in I_{1}≜\left[t_{\min},t_{\min}+t_{\Delta }\left(1-\epsilon \right)\right]$ and zero otherwise.

Let $t^{\left(1,1\right)}$ denote the expectation of $T^{\left(1,1\right)}$, and we have:

(17) $$\begin{array}{cc} t^{\left(1,1\right)}= & \int_{t\in I_{1}}t\;f_{T^{\left(1,1\right)}|N>0}\left(t\right)\;dt\\ = & \frac{1}{\lambda_{1}\alpha_{1}}+\frac{t_{1,1}^{-}-\exp\left(-\lambda_{1}\right)t_{1,1}^{+}}{1-\exp(-\lambda_{1})}\\ = & t_{\min}+t_{\Delta }\left(1-\epsilon \right)\left(\frac{1}{\lambda_{1}}-\frac{1}{\exp(\lambda_{1})-1}\right). \end{array}$$

#### 5.2.2. One-Hop Transmission Distance for the Intra-Lane Scenario

It is the vehicle that can achieve the minimum timer value $T^{\left(1,1\right)}=\min\left\{T_{1},\cdots ,T_{N}\right\}$ that will be selected as the elected backbone vehicle node. The random variables $T^{\left(1,1\right)}$ and $D^{\left(1,1\right)}$ are directly related by Equation (11). Therefore, we have:

(18) $$D^{\left(1,1\right)}=R\left(1-\frac{T^{\left(1,1\right)}-t_{\min}}{t_{\Delta }\left(1-\epsilon \right)}\right).$$

Then, the CDF of the random variable $D^{\left(1,1\right)}$, conditional on there being at least one vehicle in the range *R*, i.e., N>0, is given by:

(19) $$\begin{array}{cc} \mathrm{Pr}\{D^{\left(1,1\right)}\leq x|N>0\}= & \mathrm{Pr}\left\{T^{\left(1,1\right)}\geq t_{\min}+t_{\Delta }\left(1-\epsilon \right)\left(1-\frac{x}{R}\right)|N>0\right\}\\ = & \frac{\exp(\lambda_{1}x/R)-1}{\exp(\lambda_{1})-1}\;,\;x\in \left[0,R\right]. \end{array}$$

Let $d^{\left(1,1\right)}$ denote the expected value of $D^{\left(1,1\right)}$. Then, we have:

(20) $$d^{\left(1,1\right)}=R\left(\frac{1}{1-\exp(-\lambda_{1})}-\frac{1}{\lambda_{1}}\right).$$

#### 5.2.3. One-Hop Timer Delay for the Inter-Lane Scenario

Different from the intra-lane scenario, in the inter-lane scenario, the timer for the receiver vehicles, considering the influence of both the inter-relay distance and the link lifetime, can be represented as:

(21) $$T^{\left(1,2\right)}=t_{\min}+t_{\Delta }\left(1-\epsilon \cdot \frac{R-D^{\left(1,2\right)}}{v_{\Delta }\cdot L_{\max}}-\left(1-\epsilon \right)\cdot \frac{D^{\left(1,2\right)}}{R}\right),$$

where $v_{\Delta }$ is the difference between the speed levels of the two lanes.

Following a similar reasoning as the one used to derive the CDF of $T^{\left(1,1\right)}$, we obtain:

(22) $$\mathrm{Pr}\left\{T^{\left(1,2\right)}>t|N=n\right\}={\left[1-\left(\alpha_{2}t+\beta_{2}\right)\right]}^{n},$$

where n>0, and $t\in [t_{\min},t_{\max}]$. Moreover, we have:

(23) $$\begin{array}{c} \alpha_{2}=\frac{v_{\Delta }L_{\max}}{\epsilon Rt_{\Delta }-\left(1-\epsilon \right)v_{\Delta }L_{\max}t_{\Delta }}\;,\;\beta_{2}=\frac{\left(\epsilon R-v_{\Delta }L_{\max}\right)t_{\Delta }-t_{\min}v_{\Delta }L_{\max}}{\epsilon Rt_{\Delta }-\left(1-\epsilon \right)v_{\Delta }L_{\max}t_{\Delta }}. \end{array}$$

Based on the total probability formula, we have the CDF of the minimum intra-lane timer delay as:

(24) $$\begin{array}{cc} \mathrm{Pr}\{T^{\left(1,2\right)}\leq t|N>0\}= & 1-\sum_{n=1}^{\infty }\mathrm{Pr}\left\{T_{\min}^{\left(1,2\right)}>t|N=n\right\}\mathrm{Pr}\left\{N=n|N>0\right\}\\ = & \frac{1-\exp\left(-\lambda_{2}\left(\alpha_{2}t+\beta_{2}\right)\right)}{1-\exp(-\lambda_{2})}. \end{array}$$

Based on the expression of the conditional CDF, the conditional pdf can be obtained as:

(25) $$f_{T^{\left(1,2\right)}|N>0}\left(t\right)=\frac{\lambda_{2}\alpha_{2}\exp\left(-\lambda_{2}\left(\alpha_{2}t+\beta_{2}\right)\right)}{1-\exp(-\lambda_{2})}.$$

for $t\in I_{2}≜\left[t_{\min},t_{\min}+t_{\Delta }\right]=\left[t_{\min},t_{\max}\right]$ and zero otherwise.

Let $t^{\left(1,2\right)}$ denote the expectation of $T^{\left(1,2\right)}$, and we have:

(26) $$t^{\left(1,2\right)}=\frac{1}{\lambda_{2}\alpha_{2}}+\frac{t_{1,2}^{-}-\exp\left(-\lambda_{2}\right)t_{1,2}^{+}}{1-\exp(-\lambda_{2})}.$$

#### 5.2.4. One-Hop Transmission Distance for the Inter-Lane Scenario

Let $D^{\left(1,2\right)}$ denote the distance between the selected relay vehicles in the inter-lane case. From Equation (21), we can formulate the relation between the one-hop timer value and the transmission distance, which can be represented as:

(27) $$D^{\left(1,2\right)}=\alpha_{D}T^{\left(1,2\right)}+\beta_{D},$$

where $\alpha_{D}=R\alpha_{2}$ and $\beta_{D}=R\beta_{2}$. $\alpha_{2}$ and $\alpha_{D}$ are positive for $\epsilon >\frac{v_{\Delta }L_{\max}}{v_{\Delta }L_{\max}+R}$. Hence, if ϵ=1/2, we can see that $\alpha_{2}>0$, $\alpha_{D}>0$ when $R>v_{\Delta }L_{\max}$.

### 5.3. One-Hop Timer Delay for the Two Lane Scenario

Since $I_{1}=\left[t_{1,1}^{-},t_{1,1}^{+}\right]$ and $I_{2}=\left[t_{1,2}^{-},t_{1,2}^{+}\right]$, where the limits are determined by parameter *ϵ*, a special case of *ϵ* is taken as an example. Suppose that ϵ=1/2, $I_{1}$ and $I_{2}$ can be divided into two sub-intervals, $I_{3}≜\left[t_{1},t_{2}\right]$ and $I_{4}≜\left[t_{2},t_{3}\right]$, where $t_{1}=t_{\min}$, $t_{2}=t_{\min}+t_{\Delta }\left(1-\epsilon /\left(2v_{\Delta }L_{\max}\right)\right)$ and $t_{3}=t_{\min}+t_{\Delta }/2$. Let $T^{\left(1\right)}$ denote the minimum timer delay conditioned on the sender vehicle being located in lane $l_{1}$.

The derivation of $T^{\left(1\right)}$ will be discussed in two cases as follows. When $t\in I_{3}$, we have:

(28) $$\begin{array}{c} \mathrm{Pr}\left\{T^{\left(1\right)}\leq t|t\in I_{3}\right\}=\mathrm{Pr}\left\{T^{\left(1,1\right)}\leq t|N>0\right\}=\frac{1-\exp\left(-\lambda_{1}\left(\alpha_{1}t+\beta_{1}\right)\right)}{1-\exp(-\lambda_{1})}. \end{array}$$

Otherwise, when $t\in I_{4}$, we have:

(29) $$\begin{array}{cc} \mathrm{Pr}\{T^{\left(1\right)}\leq t|t\in I_{4}\}= & 1-\mathrm{Pr}\{T^{\left(1\right)}>t|t\in I_{4}\}\\ = & 1-\mathrm{Pr}\left\{T^{\left(1,1\right)}>t\right\}\cdot \mathrm{Pr}\left\{T^{\left(1,2\right)}>t\right\}\\ = & 1-\left(1-\mathrm{Pr}\left\{T^{\left(1,1\right)}\leq t\right\}\right)\cdot \left(1-\mathrm{Pr}\left\{T^{\left(1,2\right)}\leq t\right\}\right), \end{array}$$

where we have not included the condition N>0 to simplify the expression of the probabilities.

Substituting the CDF of $T^{\left(1,1\right)}$ and $T^{\left(1,2\right)}$ into the above equations and taking the derivate, we can obtain the pdf of $T^{\left(1\right)}$ in case $t\in I_{4}$ as:

(30) $$\begin{array}{cc} f_{T^{\left(1\right)}|t\in I_{4}}\left(t\right)= & \frac{\lambda_{1}\alpha_{1}\exp\left(-\lambda_{1}\left(\alpha_{1}t+\beta_{1}\right)\right)}{1-\exp(-\lambda_{1})}+\frac{\lambda_{2}\alpha_{2}\exp\left(-\lambda_{2}\left(\alpha_{2}t+\beta_{2}\right)\right)}{1-\exp(-\lambda_{2})}\\ - & \frac{\lambda_{1}\alpha_{1}\exp\left(-\lambda_{1}\left(\alpha_{1}t+\beta_{1}\right)\right)+\lambda_{2}\alpha_{2}\exp\left(-\lambda_{2}\left(\alpha_{2}t+\beta_{2}\right)\right)}{\left[1-\exp\left(-\lambda_{1}\right)\right]\left[1-\exp\left(-\lambda_{2}\right)\right]}\\ + & \frac{\left(\lambda_{1}\alpha_{1}+\lambda_{2}\alpha_{2}\right)\exp\left(-\left(\left(\lambda_{1}\alpha_{1}+\lambda_{2}\alpha_{2}\right)t+\left(\beta_{1}+\beta_{2}\right)\right)\right)}{\left[1-\exp\left(-\lambda_{1}\right)\right]\left[1-\exp\left(-\lambda_{2}\right)\right]}. \end{array}$$

Let $t^{\left(1\right)}$ denote the expectation of $T^{\left(1\right)}$. Then, we have:

(31) $$\begin{array}{cc} t^{\left(1\right)}= & \int_{t\in I_{3}}t\cdot f_{T^{\left(1\right)}|t\in I_{3}}\left(t\right)\;dt+\int_{t\in I_{4}}t\cdot f_{T^{\left(1\right)}|t\in I_{4}}\left(t\right)\;dt\\ =- & \frac{1}{C_{1}}\left(t+\frac{1}{\lambda_{1}\alpha_{1}}\right)\exp\left(-\lambda_{1}\left(\alpha_{1}t+\beta_{1}\right)\right){|}_{t_{1}}^{t_{3}}\\ + & \left(\frac{1}{C_{1}C_{2}}-\frac{1}{C_{2}}\right)\left(t+\frac{1}{\lambda_{2}\alpha_{2}}\right)\exp\left(-\lambda_{2}\left(\alpha_{2}t+\beta_{2}\right)\right){|}_{t_{2}}^{t_{3}}\\ + & \frac{1}{C_{1}C_{2}}\left(t+\frac{1}{\lambda_{1}\alpha_{1}}\right)\exp\left(-\lambda_{1}\left(\alpha_{1}t+\beta_{1}\right)\right){|}_{t_{2}}^{t_{3}}\\ - & \frac{1}{C_{1}C_{2}}\left(t+\frac{1}{\left(\lambda_{1}\alpha_{1}+\lambda_{2}\alpha_{2}\right)}\right)\exp\left(-\left(\left(\lambda_{1}\alpha_{1}+\lambda_{2}\alpha_{2}\right)t+\left(\beta_{1}+\beta_{2}\right)\right)\right){|}_{t_{2}}^{t_{3}}. \end{array}$$

Based on the aforementioned definition, the obtained value of $t^{\left(1\right)}$ represents $E[T_{L}]$ under the condition that the sending vehicle node is located in lane $l_{1}$ and that there is at least one vehicle in the transmission range of the sending node, i.e., N>0. In a similar way, we can obtain the probability density function and the mean value of the random variable $T^{\left(2\right)}$, defined as the minimum timer delay conditioned on the sender vehicle being located in lane $l_{2}$.

### 5.4. One-Hop Distance for the Two-Lane Scenario

Different from the derivation of the one-hop timer delay, the calculation of distance is based on the lane state transition probability. This is because neither a maximum nor minimum value of $D^{\left(1,1\right)}$ and $D^{\left(1,2\right)}$ can represent $D^{\left(1\right)}$ based on Equation (7). Let $p_{ij}$ denote the lane state transition probability from the current relay in lane $l_{i}$ to the next hop relay in lane $l_{j}$. On the basis of this definition, we can obtain that the CDF of $D^{\left(1\right)}$ as:

(32) $$\mathrm{Pr}\left\{D^{\left(1\right)}\leq d\right\}=p_{11}\mathrm{Pr}\left\{D^{\left(1,1\right)}\leq d\right\}+p_{12}\mathrm{Pr}\left\{D^{\left(1,2\right)}\leq d\right\},$$

where equations analogous to those given above hold for the CDF random of the variable $D^{\left(2\right)}$, and the derivations of $p_{ij}$ will be introduced as follows.

**Figure 4:**
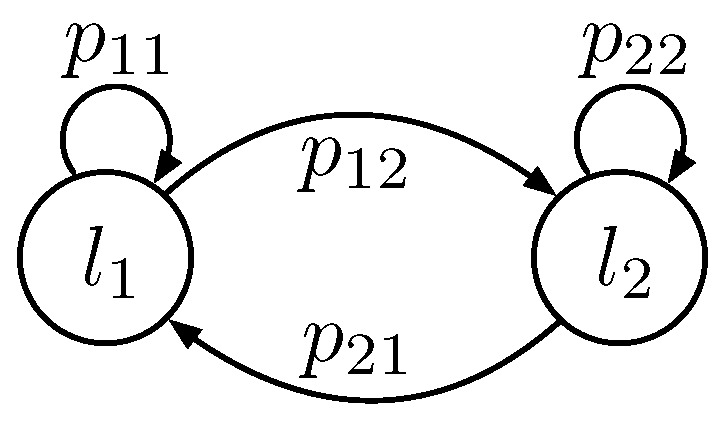
Two-lane state Markov chain transitions.

In the two-lane scenario, the transition probability matrix for lane state $\left\{X_{n},n>0\right\}$ contains four items (Figure 4), that is:

(33) $$P=\left[\begin{array}{cc} p_{11} & p_{12}\\ p_{21} & p_{22} \end{array}\right],$$

where the transition probability $p_{11}$ can be calculated out by:

(34) $$\begin{array}{cc} p_{11}= & \mathrm{Pr}\{X_{k+1}=l_{1}|X_{k}=l_{1}\}\\ = & \int \mathrm{Pr}\{T^{\left(1,1\right)}=t,T^{\left(1,2\right)}>t|T^{\left(1\right)}=t\}\;dt\\ = & \int \frac{\mathrm{Pr}\{T^{\left(1,1\right)}=t,T^{\left(1,2\right)}>t\}}{\mathrm{Pr}\{T^{\left(1\right)}=t\}}\;dt\\ = & \int \frac{\mathrm{Pr}\left\{T^{\left(1,1\right)}=t\right\}\left(1-\mathrm{Pr}\left\{T^{\left(1,2\right)}\leq t\right\}\right)}{\mathrm{Pr}\{T^{\left(1\right)}=t\}}\;dt. \end{array}$$

Substituting the results found in Section 5.3, $p_{11}$ can be represented as:

(35) $$\begin{array}{cc} & p_{11}=\int_{t\in I_{3}}\frac{f_{T^{\left(1,1\right)}|t\in I_{3}}\left(t\right)}{f_{T^{\left(1\right)}|t\in I_{3}}\left(t\right)}\;dt\;+\int_{t\in I_{4}}\frac{f_{T^{\left(1,1\right)}|t\in I_{4}}\left(t\right)\left(1-\mathrm{Pr}\left\{T^{\left(1,2\right)}\leq t|t\in I_{4}\right\}\right)}{f_{T^{\left(1\right)}|t\in I_{4}}\left(t\right)}\;dt. \end{array}$$

The transition probability can be evaluated by numerical integration of the expression in Equation (35). Specifically, the integration space $I_{3}⋃I_{4}=\left[t_{1},t_{3}\right]$ is divided into $n_{I}$ equally-sized intervals, of length $\epsilon_{t}=\left(t_{3}-t_{1}\right)/n_{I}$. Then, the integral reduces to a sum. The other three probability items, $p_{12},p_{21}$ and $p_{22}$, can be obtained with a similar method.

Based on the properties of SMP [60], we can obtain the stationary distribution of SMP. It can be deduced that the embedded Markov chain $\left\{X_{n},n\geq 0\right\}$ of the semi-Markov process $\left\{Z_{t},t\geq 0\right\}=\left\{X_{n},Y_{n},n\geq 0\right\}$ is irreducible, and the first arrival time is finite. Then, the stationary distribution of $\left\{X_{n},n\geq 0\right\}$ exists, which is denoted as $\tilde{\pi }=\left[{\tilde{\pi }}_{1},{\tilde{\pi }}_{2}\right]$. Let $\pi_{i}$ denote the result of limit $\lim_{t\to \infty }\mathrm{Pr}\{Z_{t}=i|Z_{0}=i_{0}\}$, and we have:

(36) $$\pi_{i}=\frac{{\tilde{\pi }}_{i}t^{\left(i\right)}}{\sum_{j=1}^{2}{\tilde{\pi }}_{j}t^{\left(j\right)}}.$$

where $t^{\left(i\right)}$ is the mean time spent into state *i*, before making a transition to the next visited state.

Based on the lane state stationary distribution probability, we have the expectation of the one-hop transmission distance as:

(37) $$E\left[D_{L}\right]=\pi_{1}d^{\left(1\right)}+\pi_{2}d^{\left(2\right)}.$$

The difference between the derivations of $E[D_{L}]$ and $E[T_{L}]$ lies in the timer setting rule. We will select the next hop relay with the minimum timer value; however, this relay vehicle might not be the furthest vehicle among the current relay’s neighbors. Therefore, we take the method proposed in Equation (37) as an alternative to obtain the expectation of $D_{L}$.

### 5.5. End-To-End Delay for One Transmission Path

Substituting Equation (10) into Equation (9), we can obtain the expected end-to-end delay $E[T^{c}]$ for one constructed routing path as:

(38) $$E\left[T^{c}\right]=d_{\mathrm{SD}}\frac{E[T_{L}]}{E[D_{L}]},$$

where the derivations of $E[T_{L}]$ and $E[D_{L}]$ are introduced in Section 5.3 and Section 5.4, respectively.

## 6. Performance Evaluation

In this section, the performance evaluation is conducted for our proposed routing protocol and corresponding analysis. Specifically, we compare LBRP with three other protocols and analyze its performance with different parameters. The proposed analytical architecture is also verified with results from Monte Carlo simulations.

### 6.1. Evaluation for the Designed Protocol

#### 6.1.1. Protocol Comparison Simulation Setup

In our simulator, vehicles move along a span of a multi-lane, bi-directional highway with length $L_{\mathrm{road}}$. The desired average overall vehicle density *ρ* is realized by feeding each lane with a flow of vehicles, with three lanes devoted to each direction. On each direction, lane flows have different intensities, such that the slow lane average density and the middle lane average density are three-times and two-times the density of the high speed lane, respectively. Vehicle micro-mobility is generated according to the Krauss car-following model [61,62], with the following parameters: the maximum speed for all vehicles on the road $v_{\max}=150$ km/h, the minimum distance gap between any two consecutive vehicles $d_{\min}=6$ m, the braking time $t_{\mathrm{break}}=1$ s, the correlation coefficient of driver speed $\alpha_{\mathrm{csi}}=0.95$, the maximum acceleration $a_{\max}=1.5$ m/s${}^{2}$ and the maximum deceleration $b_{\max}=3$ m/s${}^{2}$. The default values of the main parameters for our simulation are shown in Table 2.

For each round of simulation, a content request is generated in one randomly-selected vehicle, which is regarded as the SUV. The target scenario for the simulation is the road surveillance service, where the SUV wants to get the video information of road segments $d_{\mathrm{SD}}$ ahead. We assume that up to three vehicles, which carry the target video content, will serve as the candidate SOVs. All of the other vehicles will serve as the candidate BVs. As it should be guaranteed that the request message can achieve at least one SOV, we define the TTL value as $\lceil 2d_{\mathrm{SD}}/R\rceil$.

The performance of our proposed LBRP algorithm is verified with three state-of-the-art schemes:

- Distance-Based Forwarding (DBF): the forwarding decision is based on maximizing the one-hop transmission distance, i.e., the timer used by DBF is given by Equation (7) with ϵ=0. By extending the one-hop transmission distance, DBF can reduce the number of hops of the content delivery path. This is the main target of many multi-hop dissemination scheme designs, originally proposed in [63] and analyzed in, e.g., [5,6]. The DBF is essentially coincident with the Contention-Based Forwarding (CBF), specified as an alternative mode of operation of the standardized GeoNetworking protocol in [64]. Moreover, the DBF concept can also be found in the beacon-based algorithms, e.g., the GPSR protocol [6].
- Random Timer Forwarding (RTF): For each hop relay vehicle selection, the timer is independent of the positions of the sender and receiver; it is drawn according to a uniform probability density on the interval $\left[t_{\min},t_{\max}\right]$ [57,65].
- Link State-aware Geographic Routing protocol (LSGR): LSGR incorporated the link state’s influence into the routing protocol design [28]. LSGR contrived a routing metric called Expected One-transmission Advance (EOA) to improve the greedy forwarding algorithm by explicitly incorporating the link state and packets advance. However, the EOA weighting parameter might not be easily estimated for the beacon-less routing protocol design. To take a suitable case for our system scenario, we assume the link state can be represented by the link duration parameter considered in our paper. For the path construction process, the neighboring vehicle that can provide the best link state will be selected as the next hop relay vehicle. Definitely, this can improve the transmission efficiency by diminishing transmission failures, while the end-to-end delay might not be guaranteed at the same time.

In the following, we first present a comparison of our proposed protocol, LBRP, with DBF, RTF and LSGR (Section 6.1). Then, we give results to assess the accuracy of the analytical model as opposed to the simulations (Section 6.2). A final wrap-up discussion of the performance evaluation results is given in Section 6.3.

#### 6.1.2. Comparison for LBRP with Three Others

**Figure 5:**
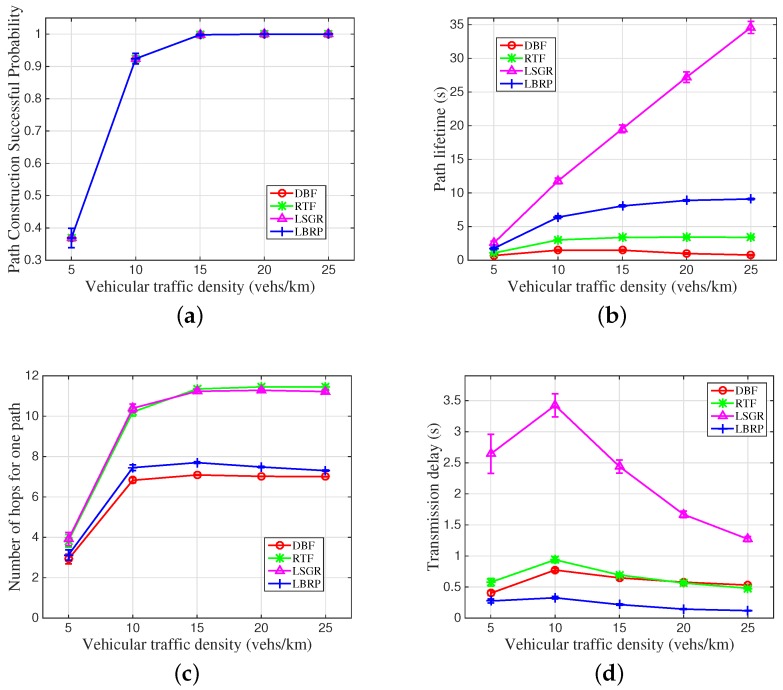
The influence of vehicular traffic density on four different routing metrics. (**a**) Path construction successful probability; (**b**) One path lifetime; (**c**) Number of hops; (**d**) Transmission delay. DBF, Distance-Based Forwarding; RTF, Random Timer Forwarding; LSGR, Link State-aware Geographic Routing.

In Figure 5, four key performance metrics are compared between our proposed LBRP and three other protocols: DBF, RTF and LSGR. In these figures, we mainly validate the influence of vehicular traffic density on performance metrics, including the path construction successful probability, the path lifetime, the number of transmission hops for one constructed path and the overall transmission delay for the target content.

#### 6.1.3. Path Construction Successful Probability

In Figure 5a, we first conduct the comparison of the path construction successful probability among the four compared routing protocols. It can be seen that all four mentioned protocols show relatively identical performance, which might be due to exactly the same vehicular topology information being used in the simulations. Moreover, with the increase of the vehicular traffic density, a higher successful probability can be obtained in constructing a multi-hop path between the SUV and the SOV. When the vehicular traffic density is higher than 15 vehicles per kilometer, the path construction successful probability approaches one as the most anticipated value.

#### 6.1.4. One Path Lifetime

In Figure 5b, we compare the performance of the constructed path’s lifetime. Recall that the path lifetime is determined by the link with the minimum lifetime. As can be seen from the figure, LSGR produces the longest path lifetime since it prefers the neighboring vehicle that can maintain high connectivity performance. Next in importance, our proposed LBRP shows the second best path lifetime as a result of the consideration of both link lifetime and one-hop transmission distance information on the protocol design. On the contrary, without the consideration of the link connectivity property, the path tends to be fragile, as illustrated in DBF and RTF. Especially for DBF, with the largest inter-relay distance, the constructed path is the most easily broken.

#### 6.1.5. Number of Hops for One Constructed Routing Path

In Figure 5c, we compare the number of hops needed for constructing a routing transmission path between the transceiver. Reasonably, LSGR produces the largest number of hops because the best connectivity property can always be found in the nearby vehicles, which might reduce the one-hop transmission distance. Similarly, RTF shows a closing tendency on the number of hops with LSGR, due to it choosing the next hop relay randomly. As expected, DBF illustrates the least number of hops since it chooses the furthest vehicle in the radio range as the relay. For our proposed LBRP, with the consideration of both speed and position information, the increase of the number of hops compared to DBF is not much. As can be seen from the simulation results, the hop count for LBRP is within one hop greater than that of DBF.

#### 6.1.6. The Whole Content Transmission Delay

In Figure 5d, we compare the whole content transmission delay issue, which is the main performance metric for the user’s satisfaction. As can be seen from this figure, our proposed LBRP can generate the smallest end-to-end delay for the whole content dissemination. For the three other protocols, without the consideration of either speed or position information, the delay performance can be affected to a certain extent. The detailed reason for the presented results lies in the previously-illustrated performance metric comparison in Figure 5a–c.

### 6.2. Evaluation for the Analytical Model

#### 6.2.1. Analysis Verification Simulation Setting

In the verification process for the proposed analytical model, we select two lanes from six lanes in the protocol verification parts as an example. Here, we choose the lanes with the slowest speed and the middle speed, where vehicles drive from west to east. Let $\rho_{1}$ and $\rho_{2}$ denote the vehicular traffic density for the slow speed driving lane and the middle speed driving lane. For the driving speed, we assume that the default average speeds are defined as 70 and 90 km/h for the two driving lanes, while the maximum driving speeds are 90 and 120 km/h for the two driving lanes, respectively. The Krauss car-following mobility model is used with identical parameters setting as that in the protocol comparison simulations.

#### 6.2.2. One-Hop Timer Delay for the Intra-Lane Scenario

**Figure 6:**
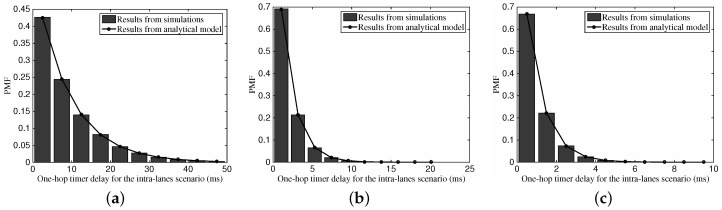
The PMF of one-hop backoff timer delay for the intra-lane scenario with different vehicular traffic density. (**a**) $\rho_{1}=10$ vehs/km; (**b**) $\rho_{1}=50$ vehs/km; (**c**) $\rho_{1}=100$ vehs/km.

Figure 6 shows the Probability Mass Function (PMF) of one-hop timer delay for the intra-lane scenario with the histogram from simulations and the curve from our proposed analytical model. As can be seen from the figures, the results from the simulations match well with those obtained via the analytical model. For the intra-lane scenario with a dedicated vehicular density, we can see that the probability value presents a declining tendency with the increase of possible one-hop delay. Moreover, from Figure 6a–c, with the increase of the vehicular traffic density $\rho_{1}$ from 10 vehs/km (vehicles per meter) to 100 vehs/km, we can see that the maximum range drops gradually from 50 ms down to 10 ms. This outcome is a consequence of the bigger number of one-hop neighboring vehicles with the vehicular traffic density, which can help the sender’s choice on the next-hop relay approaching the optimal one.

#### 6.2.3. One-Hop Transmission Distance for the Intra-Lane Scenario

**Figure 7:**
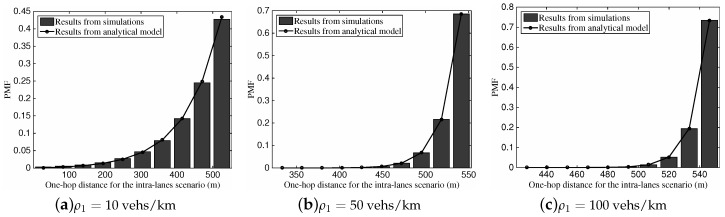
The PMF of one-hop transmission distance for the intra-lane scenario with different vehicular traffic density.

With the identical set of parameters, Figure 7 depicts the one-hop transmission distance’s PMF versus different vehicular traffic density. Similar to the results in Figure 6, we can see that the analysis results are consistent with the simulation results. The PMF values show an upward tendency with the increase of the one-hop distance, which is motivated by the fact that a longer relaying distance is preferred for the intra-lane scenario. Again, with the rise of vehicular traffic density and corresponding more neighboring vehicles, the larger one-hop transmission distance can be obtained.

#### 6.2.4. One-Hop Timer Delay for Inter-Lane Scenario

**Figure 8:**
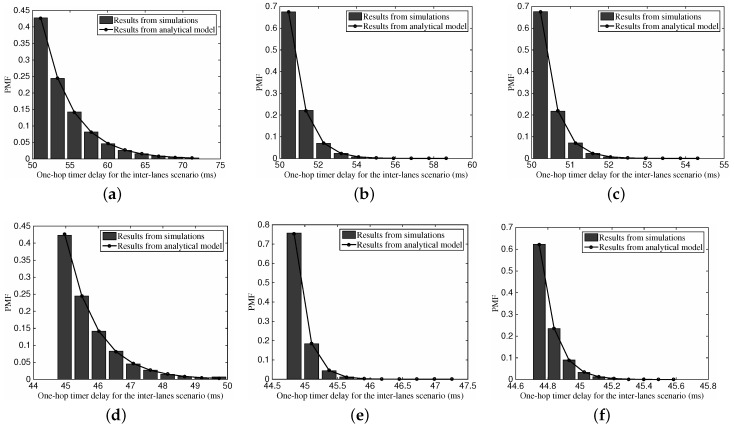
The PMF of one-hop timer delay for the inter-lane scenario with different vehicular traffic density: (**a**) $\rho_{2}=10$ vehs/km, $v_{\Delta }=40$ km/h; (**b**) $\rho_{2}=50$ vehs/km, $v_{\Delta }=40$ km/h; (**c**) $\rho_{2}$ =100 vehs/km, $v_{\Delta }=40$ km/h; (**d**) $\rho_{2}=10$ vehs/km, $v_{\Delta }=20$ km/h; (**e**) $\rho_{2}=50$ vehs/km, $v_{\Delta }=20$ km/h; (**f**) $\rho_{2}$ = 100 vehs/km, $v_{\Delta }=20$ km/h.

Figure 8 depicts the PMF of one-hop timer delay for the inter-lane scenario with different vehicular traffic density $\rho_{2}$ and speed difference $v_{\Delta }$. For the comparison among Figure 8a–c, we can see that the higher one-hop timer delay can be obtained with the larger speed difference. This is because the link between vehicles gets increasingly more vulnerable when the speed difference grows. In addition, with the increase of vehicular density, the one-hop timer delay distribution tends to be compact, which is because the chosen relay vehicle might be of similar characteristics with more candidates.

#### 6.2.5. One-Hop Transmission Distance for the Inter-Lane Scenario

**Figure 9:**
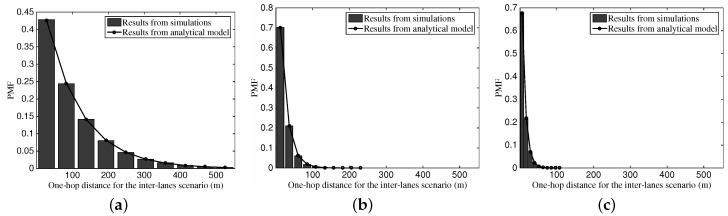
The PMF of one-hop transmission distance for the inter-lane scenario with different vehicular traffic density: (**a**) $\rho_{2}=10$ vehs/km; (**b**) $\rho_{2}=50$ vehs/km; (**c**) $\rho_{2}=100$ vehs/km.

Figure 9 gives the comparison between the one-hop transmission distance’s distribution from simulations and analysis in the inter-lane scenario. Again, the analytical model can illustrate the realistic distribution properties of the one-hop transmission distance in the inter-lane scenario. Different from the distribution in Figure 9, we can see that the one-hop distance shows a declining tendency.

#### 6.2.6. One-Hop Timer Delay for the Two-Lane Scenario

**Figure 10:**
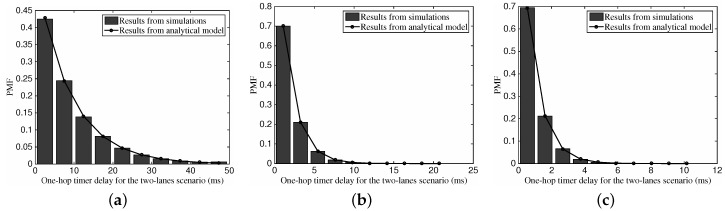
The PMF of one-hop timer delay for the two-lane scenario with different vehicular traffic density, where $v_{\Delta }=20$ km/h. (**a**) $\rho_{1}=10,\;\rho_{2}=5$ vehs/km; (**b**) $\rho_{1}=50,\;\rho_{2}=25$ vehs/km; (**c**) $\rho_{1}\;=\;100$, $\rho_{2}=50$ vehs/km.

Figure 10 illustrates the influence of different vehicular traffic density on the one-hop timer delay in the two-lane scenario. In this figure, we use the case of both lanes are of identical vehicular traffic density, which can refers to cases with non-identical *ρ*. Again, the results from the analysis accord with those from the simulations, which verifies that our analytical model works also in the two-lanes scenario. With the increase of the vehicular density, we can also observe that the distribution shape tends to be more compact.

#### 6.2.7. One-Hop Transmission Distance for the Two-Lane Scenario

**Figure 11:**
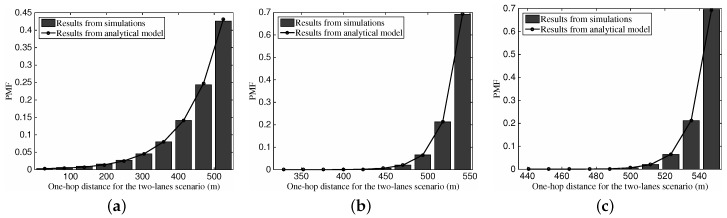
The PMF of one-hop transmission distance for the two-lane scenario with different vehicular traffic density, where $v_{\Delta }=20$ km/h. (**a**) $\rho_{1}=10,\;\rho_{2}=5$ vehs/km; (**b**) $\rho_{1}=50$, $\rho_{2}=25$ vehs/km; (**c**) $\rho_{1}$ = 100, $\rho_{2}$ = 50 vehs/m.

Figure 11 depicts the PMF of one-hop transmission distance for the two-lane scenario with different vehicular traffic density. Once again, we can find the accuracy of our analytical model on describing the distribution of the one-hop transmission distance in the two-lane scenario. With the defined values of default parameters, we can see that the one-hop transmission distance tends to approach the radio range *R*. With the increase of the vehicular traffic density, the PMF shape becomes more compact.

### 6.3. Discussion

In this section, we have evaluated the performance of our proposed protocol, LBRP, for the multi-hop large volume content transmission, and verified the accuracy of our analytical model for the calculation of the probability distribution and moments of the back-off timer delay. Even a simple scenario as the one considered in this performance evaluation is enough to highlight the benefit brought about by accounting for link lifetime besides the hop length in the construction of the multi-hop chain of BVs. The key point is that we shift the emphasis of the performance metric on the overall content transmission time, which is a key performance indicator closer to user experience and hence valuable to assess the overall acceptability of the protocol. While a single path made of less hops is the furthest possible next BV searched for, path breakdowns are then more frequent due to vehicle mobility. Hence, the importance of accounting explicitly for the expected link lifetime when setting up the link chain of the path. This is achieved by LBRP only using information known to vehicles or carried via the same signaling message that is used to elect the chain of BVs. No beacons or pre-acquired maps or infrastructure nodes (RSUs) are required to support LBRP, which make it suited for an easy deployment, provided vehicles are equipped with DSRC devices (or any equivalent communication interface that enables direct proximity communication in the range of a few hundred meters, e.g., millimeter waves, VLC, LTE D2D).

Note that the LBRP has one parameter *ϵ* that balances the importance on the back-off timer delay between the link transmission distance and link lifetime. Given the deterministic network topology, we can find a balance between these two metrics, which cannot easily be achieved in a dynamic network. In the simulator, we set the value of *ϵ* as 0.5 in advance for both protocol evaluation and model verification. This is not necessarily an optimal value for each network topology; nevertheless, we can see that LBRP outperforms the other three selected ones. As future work, we will continue to find the optimal strategy for *ϵ* in each possible network topology, including more road networks.

## 7. Conclusions

Considering the great overhead on beacon information exchange in the intermittent vehicular networks, a Lifetime-aware Beacon-less Routing Protocol (LBRP) is proposed. Instead of continuous information exchange with the neighbor vehicles, each vehicle makes the forwarding decision based on the message header information and its current state, including the speed and position information. Based on the concise information, all of the receivers will set a timer, and the one that first counts down to zero will be selected as the next hop relay. The timer setting is determined by the one-hop transmission distance and the link lifetime value. Moreover, an analytical architecture is proposed to give an expected timer value for one constructed routing path. LBRP is verified with extensive simulations to show a greater system performance, especially a lower end-to-end delay. For the not fully-connected vehicular networks, e.g., in the night, an infrastructure-based protocol should be selected as a candidate way, which will be our next step of the work.

## Figures and Tables

**Table 1:** Symbols definitions. SOV, SOurce Vehicle; SUV, SUbscribing Vehicle; BV, Backbone Vehicle.

Symbol	Definition
*R*	the radio range for transmission between vehicles
$T_{i}^{c}$	the consumed time for the *i*-th control sub-phase
$T_{i}^{d}$	the consumed time for the *i*-th data transmission sub-phase
$T_{e2e}$	the overall content transmission time
$T_{\mathrm{data}}$	the useful data delivery time
$N_{p}$	the number of transmission phases
*V*	the amount of data to be delivered
*C*	the link bit rate of physical layer
$L_{i,j}$	the holding time for the *j*-th link of the *i*-th phase
$s_{i,j}\left(t\right)$	the RSS of the *j*-th link in the *i*-th phase at time *t*
$s_{\mathrm{th}}$	the minimum RSS threshold for maintaining the radio connection
$H_{i}$	the number of hops for the *i*-th transmission phase
$T_{L_{i,j}}$	the delay for the *j*-th link in the *i*-th transmission phase
$T_{L}$	the backoff timer delay for a general link
$w_{k}$	the weight for the decision on the *k*-th candidate SOV
$d_{k}^{0}$	the initial inter-vehicle distance between SUV and the *k*-th candidate SOV
$t_{\min},t_{\max}$	the minimum and maximum backoff timer values
$t_{\Delta }$	The maximum backoff timer difference
*D*	a general representation of the inter-vehicle distance
$D_{\max}$	the maximum radio range (defined as *R* in this paper)
$L_{\max}$	the maximum link lifetime defined for the backoff timer calculation
*ϵ*	the weighting ratio between link lifetime and transmission distance
$\rho_{i}$	the traffic density for the *i*-th road lane (vehicles per meter)
$v_{i}$	the expected speed for the *i*-th road lane (meters per second)
$\lambda_{i}$	the average number of one-hop neighboring vehicles for the *i*-th lane
$l_{k}$	the *k*-th road lane with increasing speed
$X_{n}$	the lane state for the *n*-th BV, which can be $l_{1}$ or $l_{2}$ for a two-lane scenario
$Y_{n}$	the backoff timer delay between two consecutive BVs
$E[T^{c}]$	the expected delay for a generic routing phase out of the $N_{p}$
E[H]	the average number of hops
$E[T_{L}]$	the expected timer delay in one V2V link
$d_{\mathrm{SD}}$	the end-to-end distance between the SUV and the selected SOV
$E[D_{L}]$	the average distance between two consecutive BVs for each link
$T^{\left(1,1\right)}$	the backoff timer delay conditional on $X_{k}=l_{1}$ and $X_{k+1}=l_{1}$
$T^{\left(1,2\right)}$	the backoff timer delay conditional on $X_{k}=l_{1}$ and $X_{k+1}=l_{2}$
$D^{\left(1,1\right)}$	the transmission distance conditional on $X_{k}=l_{1}$ and $X_{k+1}=l_{1}$
$T_{k}^{\left(1,1\right)}$	the backoff timer delay for the *k*-th candidate vehicle given $X_{k}=X_{k+1}=l_{1}$
$t^{\left(1,1\right)}$	the expectation of random variable $T^{\left(1,1\right)}$
$v_{\Delta }$	the difference between the speed levels of the two lanes
$T^{\left(1\right)}$	the minimum backoff timer delay given the sender vehicle locating in lane $l_{1}$
*P*	the lanes state transition probability matrix
$p_{ij}$	the transition probability of message delivery from vehicle on $l_{i}$ to vehicle on $l_{j}$

**Table 2:** The default values of the main parameters in our simulations. LBRP, Lifetime-aware Beacon-less Routing Protocol.

Parameter	Description	Value
$t_{\min}$	the minimum timer	0 ms
$t_{\max}$	the maximum timer	100 ms
*ϵ*	the parameter for the proposed LBRP	0.5
$L_{\max}$	the link lifetime threshold	10 s
*V*	the target content size	5 MB
$P_{t}$	the transmission power	500 mW
*C*	the data transmission rate	3 Mbps
*R*	the radio coverage range	558 m
$d_{\mathrm{width}}$	the width of each driving lane	4 m
$d_{\mathrm{SD}}$	the inter-transceiver distance	2 km
$L_{\mathrm{road}}$	the length of the considered highway span	20 $R_{\max}$
Δt	the minimum sampling time interval	1 s
$\rho_{1}$	the vehicle density for the high-speed lane	10 vehicles/km
$v_{\max}$	the maximum speed for all vehicles on the road	150 km/h
$d_{\min}$	the minimum distance gap between any two vehicles	6 m
$t_{\mathrm{break}}$	the time needed for braking	1 s
$\alpha_{\mathrm{csi}}$	the correlation coefficient of driver speed	0.95
$a_{\max}$	the maximum acceleration	1.5 m/s${}^{2}$
$b_{\max}$	the maximum deceleration	3 m/s${}^{2}$
